# Damaged mitochondria in Fanconi anemia – an isolated event or a general phenomenon?

**DOI:** 10.18632/oncoscience.29

**Published:** 2014-04-21

**Authors:** Giovanni Pagano, Pavithra Shyamsunder, Rama S. Verma, Alex Lyakhovich

**Affiliations:** ^1^ Italian National Cancer Institute, G Pascale Foundation, CROM, Mercogliano, AV, Italy; ^2^ Stem Cell and Molecular Biology laboratory, Department of Biotechnology, Indian Institute of Technology Madras, Chennai; ^3^ Duke-NUS Graduate Medical School, Singapore; ^4^ Novosibirsk Institute of Molecular Biology and Biophysics, Russian Federation; ^5^ Queen's University Belfast, UK

**Keywords:** Fanconi anemia, mitochondrial dysfunction, oxidative stress, reactive oxygen species, DNA damage and repair

## Abstract

Fanconi anemia (FA) is known as an inherited bone marrow failure syndrome associated with cancer predisposition and susceptibility to a number of DNA damaging stimuli, along with a number of clinical features such as upper limb malformations, increased diabetes incidence and typical anomalies in skin pigmentation. The proteins encoded by FA-defective genes (FANC proteins) display well-established roles in DNA damage and repair pathways. Moreover, some independent studies have revealed that mitochondrial dysfunction (MDF) is also involved in FA phenotype. Unconfined to FA, we have shown that other syndromes featuring DNA damage and repair (such as ataxia-telangiectasia, AT, and Werner syndrome, WS) display MDF-related phenotypes, along with oxidative stress (OS) that, altogether, may play major roles in these diseases. Experimental and clinical studies are warranted in the prospect of future therapies to be focused on compounds scavenging reactive oxygen species (ROS) as well as protecting mitochondrial functions.

Independent studies have identified MDF in FA [1-6], an inherited bone marrow failure (BMF) syndrome associated with DNA damage and repair (DDR) pathways, along with susceptibility to non-lymphocytic leukemias and other malignancies, and other clinical complications such as diabetes and malformations [7,8]. FA represents a unique model disorder that raised general attention in the last decade since it was discovered that one of the encoded proteins by the FA subgroup D1 (FANCD1) was identical with the breast cancer-related BRCA2 gene [9]. The current state of knowledge on FA pathway relies on at least 16 genes corresponding to the FA genetic subgroups FA-A, -B, -C, -D1, -D2, -E, -F, -G, -I, -J, -L, -M, -N, -O, -P and -Q [8,10]. When any of those genes is biallelically mutated, except for the X-linked FANCB, the FA disease occurs. The FA pathway is recognized to protect and regulate DNA from interstrand crosslinks [10-12]. Most of the mutations in the FA pathway inactivate a nuclear FA core complex, consisting of proteins FANCA, -B, -C, -E, -F, -G, -L, and -M and at least four FA-associated proteins, FAAP16, FAAP20, FAAP24, and FAAP100. The main known function of the FA core complex is to monoubiquitinate chromatin complex of two other FA proteins, FANCD2 and FANCI upon DNA damage [13-15]. Inactivation of the FA core complex does not allow monoubiquitination of FANCD2-FANCI, leading to a defect in downstream DNA repair signaling, consisting of FANCD1/BRCA2, FANCJ/BRIP1/BACH1, FANCN/PALB2, FANCO/SLX4, and FANCP/RAD51C. The ubiquitinated FANCD2 recruits ubiquitin zinc finger domain-containing DNA repair proteins such as FAN1, FANCP (SLX4), TLS polymerases eta and finally mediates DNA homologous recombination together with RAD51 and BRCA1 [16-24].

Another line of studies, dating back to 1980's, has provided consistent evidence for a role of OS in FA phenotype, such as excess oxygen sensitivity [25-27], in vitro and in vivo accumulation of oxidative DNA damage [28,29], and other anomalies of redox endpoints [30]. Most notably, direct implications of FANC proteins in redox pathways have been reported. The FANCC protein was found to be associated with redox-related activities, namely NADPH cytochrome P450 reductase [31,32] and GST [32]. The FANCG protein interacts with a P450 protein, cytochrome P450 2E1 (CYP2E1) [34], an activity also known to be involved in redox biotransformation of xenobiotics including, e.g., MMC [35,36]. The FANCA and FANCG proteins were found to respond to redox state in terms of physical structure related to their ability to form disulphide bonds in the FA protein complex. Thus, FANCA, FANCC and FANCG were found to interact with redox state, also accounting for excess MMC sensitivity [31-37]. A set of independent studies showed implications of BRCA1 (FANCD2) with OS. Dziaman et al. reported excess oxidative DNA damage in breast and ovary cancer patients with defective BRCA1 vs. cancer-free BRCA1 carriers and vs. control donors [38]. Another study by Li et al. showed functional interaction of FANCD2 and the forkhead transcription factor forkhead box O 3a (FOXO3a), which colocalized with FANCD2 foci in response to OS; the authors suggested that interacting FANCD2/FOXO3a contribute to cellular antioxidant defense [39,40].

Consistent with the links of FA phenotype – and of FA proteins - with OS, and given the well-established relationships between redox pathways and MDF, a set of independent studies revealed that mitochondria are actually involved in FA phenotype, from the observation that FANG localizes to mitochondria [2]. Major mitochondrial functions were found significantly altered in FA cells of genetic subtypes A, C, D2 and G, namely ATP production, mitochondrial membrane potential (ΔΘ), mitochondrial ultrastructure, defective mitochondrial peroxiredoxin-3, and oxygen consumption [1-3]; these malfunctions were not found in corrected FA cells. Another study, conducted on transcripts from bone marrow cells from FA patients vs. healthy donors, found that genes involved in mitochondrial bioenergetic pathways, i.e. Krebs cycle and electron transport chain were significantly down-regulated, approximately by 1.5- to 2-fold [4]. These findings, both arising from freshly drawn bone marrow cells and from lymphoblastoid cells or fibroblasts, point to an in vivo occurrence of MDF in FA patients, unconfined to FA cell cultures [1-4].

A possible scenario may be suggested for FA-associated MDF and OS: normal cell conditions undermine that mitochondria actively synthesize ATP (State 3) and the rate of electron transport is accelerated upon transferring ADP, phosphate and protons across the inner membrane. In that state almost 90% of oxygen is consumed by the respiratory chain and is reduced to water. One may assume that oxidative damage is accumulated in FA cells thus resulting in MDF and affecting both ATP production and cellular respiration. This state moves the majority of FA mitochondria toward semi-resting state (State 4), where ATP production is defective and the rate of oxygen consumption is low. All these events may result in mitochondrial abnormalities [1]. Our recent data, from six FA patients as reported in Appendix I, showed down-regulation of several mitochondrial genes in cells from FA patients, confirming an involvement of MDF in FA phenotype (Fig. 1). Among those genes, nicotinamide nucleotide transhydrogenase (NNT) may play a role in detoxifying ROS as it was found that NNT knockdown resulted in impaired redox potential and increased ROS levels [41]. NNT may control ROS level and cellular redox state by replenishment of GSH antioxidant systems and mitochondrial repair enzymes (thioredoxin, glutaredoxin, peroxiredoxins and phospholipid hydroperoxidase) and contribute to maintainence of the mitochondrial membrane potential through generation of a proton gradient [42,43].

**Figure 1:**
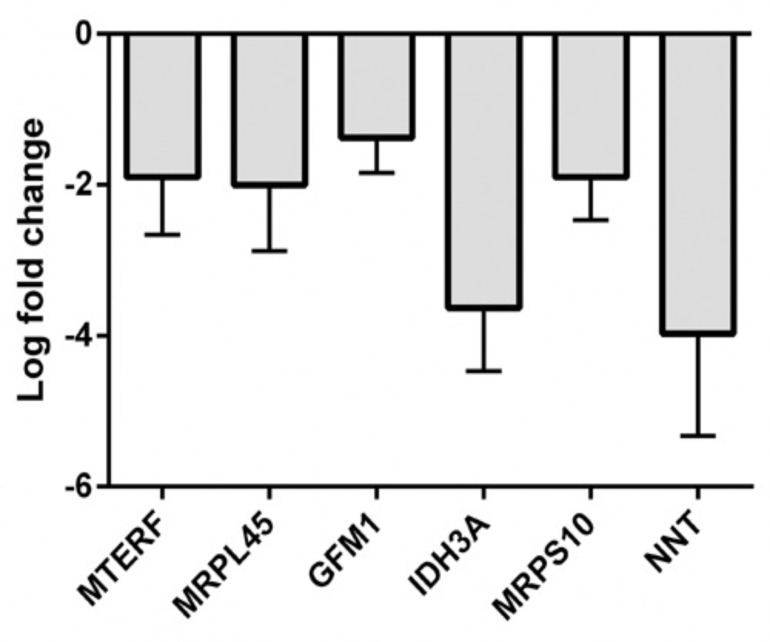
Downregulation of mitochondrial genes in FA patients Total RNA isolated from peripheral blood of 6 Fanconi anemia patients from Andhra Mahila Sabha Hospital, Chennai, or from individuals with no symptoms of FA, was amplified using Express Art mRNA amplification kit micro version (Artus GmbH, Germany), labeled with Cy3 Post-Labeling Reactive Dye Pack (GE Healthcare UK limited, UK), fragmented and purified using Express Art Amino allyl mRNA amplification kit and YM10 columns (Millipore, USA). 10.0 mg of the labeled amplified RNA was used for hybridization with the Human 40K (A+B) OciChip array. Hybridization was performed using automated hybstation HS 4800. Hybridized chips were scanned using Affymetrix 428TM array scanner at three different PMT gains. Differentially expressed genes were filtered and the results represent the most downregulated mitochondrial genes. A threshold log fold change (LFC) of 3.0 was fixed to attain FDR of less than 0.05.

An involvement of OS and MDF in FA phenotype, far from being unique, is recognized for other disorders, including mitochondrial and other genetic diseases, as well as an extensive number of diseases pertaining to a broad range of medical disciplines, and involving mitochondrial damage to cells of, e.g., brain, heart, liver, blood, kidney, lung, and eye, as reviewed recently [5,6,44,45]. Table 1 shows a selection of cancer-prone and/or progeric genetic diseases, suggesting that they share clinical and biochemical features both involving defective DNA repair (DDR), and revealing a direct evidence of MDF/OS, including altered mitochondrial functions and/or ultrastructure, higher ROS levels and imbalance of cellular bioenergetics pathways. Interestingly, many of the mitochondrial-related diseases (MRD) show involvement of DDR pathways (either at mtDNA or at nuclear DNA level). Altogether, this allows us to suggest a simplified scheme (Fig. 2), where ROS accumulated in DDR may equally affect and damage mitochondria and - at the same time – defects in mitochondria may provoke accumulation of ROS followed by OS and DNA damage. In other terms, in spite of different origins, these two classes of diseases may contribute to common – or analogous - phenotypes.

**Table 1:** DDR-related diseases have elevated ROS and share phenotypes with mitochondria-related disorders (MRD)

DDR disease	Phenotypes common for MRD	ROS and mitochondrial involvement	Ref.
Ataxia-Telangiectasia (A-T or Louis–Bar syndrome)	Impaired immunity, increased incidence of cancer, delayed onset or incomplete pubertal development, early menopause, slowed rate of growth, dysarthria, diabetes, premature changes in hair and skin;	Intrinsic mtDNA repair defects; mitochondrial requirement for ATM activation by extranuclear OS;	45-48
Bloom syndrome (BS or Bloom–Torre–Machacek syndrome)	Deficiency in certain immunoglobulin classes, hypogonadism, premature cessation of menses, chronic lung problems, diabetes, and learning disabilities, mental retardation;	Increased ROS production, mutations in energy metabolism gene PKM2, loss of mitochondrial membrane potential.	49-51
de Barsy syndrome	Musculoskeletal, neurological abnormalities, cataracts, short stature, dystonia, premature aging	mutations in mitochondrial enzyme PYCR1	52
Cockayne syndrome	Growth failure, impaired development of the nervous system, photosensitivity, premature aging, hearing loss and eye abnormalities	Deficiency in mitochondrial repair of 8-oxoguanine; Cockayne syndrome (B) protein promotes mtDNA stability; high ROS level;	53-56
Cerebral palsy (CP)	Disorders of the development of movement, epilepsy, apraxia, dysarthria, intellectual and learning disabilities, urinary incontinence, metabolic and cognitive dysregulation	Sensitivity to ROS,mitochondrial myopathies due to NADH dehydrogenase deficiency, generation of superoxide;	57-59
Cornelia de Lange syndrome (CdLS)	Growth and mental retardation, gastrointestinal disorders, brain abnormalities and hypertrophic cardiomyopathy;	Mutated mitochondrial ribosomal protein MRPS22, OXPHOS complex I, III and IV deficiency;	60
Fanconi anemia (FA)	Growth retardation, diabetis, metabolic disorders, immunoresponse impairment	Some FA proteins are localized in mitochondria; high ROS and damaged mitochondria; accumulation of oxidized proteins in FA cells;	1-6, 25-40,61,62
Friedreich's ataxia	Loss of coordination, vision and hearing impairment, diabetes, heart disorders	Deficiency of a key encoded protein frataxin leads to mitochondrial iron overload;	63
Li–Fraumeni syndrome	Several kinds of cancer are involved;	Increased oxidative metabolism	64,65
Von Hippel-Lindau	Headaches, vision problems, high blood pressure, hyperglycemia	VHL may contribute to tumorigenesis through mitochondria-based action, stimulates mitochondrial oxidative phosphorylation complex biogenesis, increased sensitivity of HIF-1α;	63-68
Ligase IV (LIG4)	Microcephaly, growth retardation, developmental delay, skin anomalies, immunodeficiency, diabetes;	Participation in mitochondrial metabolism; the key encoded protein Tdp1 participates in the repair of mt DNA	69-70
Nijmegen breakage syndrome (NBS)	Microcephaly, short stature, immunodeficiency;	Increased OS, defect in mitochondrial p53 accumulation;	71
Retinoblastoma (Rb)	Deterioration of vision, faltering growth or delayed development;	Rb protein induces apoptosis directly at the mitochondria	72,73
Spinocerebellar Ataxia (SCAE)	Epilepsy	Mitochondria-mediated cell degeneration, MDF, OS	74,75
Severe combined immunodeficiency (SCID)	Defective antibody response, severe bacterial, viral, or fungal infections, lung disease	Mitochondrial adenylate kinase 2 malfunction	76
Tuberous sclerosis complex (TSC)	Cardiac rhabdomyomas, epilepsy, mental retardation and autism, brain lesions;	Loss of Tsc1 is linked to MDF. Tdp1, a TSC gene, participates in the repair of mtDNA	77,78
Xeroderma pigmentosum (XP)	Diabetes mellitus, variable immune deficiency;	Abnormal ultrastructural changes in mitochondria, OS and MDF;	79,80
Wilms' tumor (nephroblastoma)	High blood pressure, diabetes insipidus	Reduced aerobic energy metabolism	81,82
Werner Syndrome (WS or progeria)	Cataracts, diabetes (type 2), heart and arterial disease	Generation of mitochondrial ROS in the absence of WRN; contribution of the WRN mutation in mitochondrial DNA to diabetes mellitus	83,84

**Figure 2:**
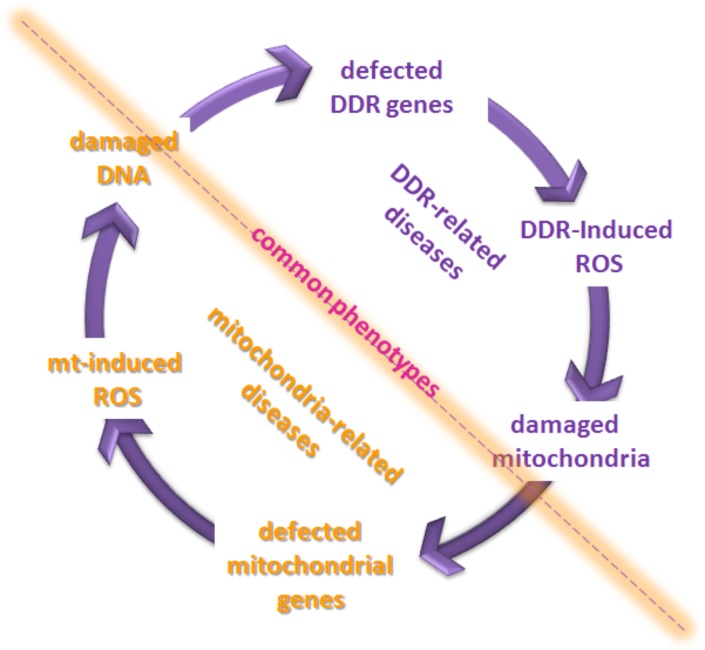
Scheme illustrating possible involvement of ROS into phenotypes of DDR and MDR diseases

It was a common stereotype that mitochondria were considered as organelles, only responsible for cellular energetic pathways. Conversely, only 3% of the genes necessary to make a mitochondrion are allocated for making ATP, whereas 97% are involved in the major metabolic pathways [85]. Mitochondria contain the rate-limiting enzymes for pyrimidine biosynthesis [85], heme synthesis [86], detoxification of ammonia in the urea cycle in the liver [87], cholesterol metabolism [88], neurotransmitter metabolism [89], free radical production and detoxification [90] and oxidative phosphorylation (OXPHOS) [91,92]. Not surprisingly, a mitochondrial basis to illness involves a number of neurologic and psychiatric disorders, malignancies, metabolic diseases, cardiovascular diseases, and autoimmune diseases [5,6,93-100].

Cancer predisposition in DDR diseases is a well-established fact and most of the DDR evolve various malignancies. Mitochondrial dysfunction has been also associated with a wide range of solid tumors, proposed to be central to the aging process, and found to be a common factor in the toxicity of a variety of xenobiotics [101]. An irreversible damage to OXPHOS leads to a shift in energy metabolism towards enhanced aerobic glycolysis in most cancers, thus mutations in mtDNA represent an early event during tumorigenesis. Due to the lack of introns, histones and limited repair mechanisms, mtDNA is more susceptible to mutations, including ROS-dependent ones. Mutations in mtDNA can contribute to the development of breast [102] and colorectal cancers [103], leukemia [104] and hepatocellular carcinoma [105]. There are many reasons to believe that ROS, acting both as mutagens and cellular mitogens, may play a role in tumor progression, thus suggesting a possible new avenue for the development of a treatment to suppress metastasis. In this regard, natural antioxidants should be considered for mitochondria-oriented FA therapy (mitochondrial nutrients, such as α-lipoic acid and coenzyme Q10) [6]. Interestingly, several compounds used in the treatment of FA patients, whose mechanisms of action in FA are largely unknown (ouabain, curcumin, androgen analogs) were also used in the treatment of MRD, e.g. heart disease (ouabain), or AD (curcumin) [106-108]. In MRD, these agents are known to inhibit Na(+)/K(+)-ATPase (ouabain), influence mitochondrial oxidation of cholesterol (oxandrolone, oxymetholone), prevent membrane permeability transition in mitochondria (thus reducing ROS by increasing glutathione) [106-112]. Therefore, it is highly suggestive that the effects of the above drugs in FA are linked to mitochondrial-related ROS. In addition to inactivating ROS by antioxidants, another strategy is to use artificial uncoupling agents that decrease proton gradient and then ROS production [113]. Unfortunately, therapeutic window(s) between efficacy and toxicity of such agents is too narrow. In order “to widen” the window between antioxidant and prooxidant concentrations, novel conjugates of plastoquinone and penetrating cations have been recently suggested [114]. Clinical studies focusing on novel ROS-scavenging compounds as well as agents preventing mitochondria from accumulation of ROS are warranted in the prospect of future therapy.

## SUPPLEMENTARY TABLE


