# Supplementary material for: Damaged mitochondria in Fanconi anemia – an isolated event or a general phenomenon?
Source: Oncoscience. 2014 Apr 21;1(4):287–95. doi: 10.18632/oncoscience.29 (PMC4278298; doi:10.18632/oncoscience.29)
Supplement: Supplementary file 1 [file oncoscience-01-0287-s001.pdf]

Annex 1

Patient samples	Age	Sex	Consanguinity	H/O FA in Family	H/O Cancer in Family	Small for date	Anaemia	Infection	Bleed	Pigmentation	Café au lait spots	Hypopigmentation
FAP1	7	M	+	-	-	+	+	-	+	+	+	+
FAP2	5	F	+	+	-	+	+	-	-	+	-	-
FAP3	9	M	+	+	-	+	+	-	-	+	+	-
FAP4	5	M	+	+	-	+	+	-	-	+	+	+
FAP5	6	M	-	+	-	-	+	+	-	+	+	+
FAP6	2	M	-	+	-	-	+	+	-	+	+	+

Patient samples	Small Featured Face	Eye Anomaly	Ear Anomaly	Absence of radial Pulse	Thumb Anomaly	Thenar Hypoplasia	Pancytopenia	Renal Anomaly	Cardiac Anomaly	Short stature	Hypogenitalia	Microcephaly
FAP1	+	+	-	-	+	+	+	+	-	+	+	+
FAP2	+	+	+	+	+	+	+	+	-	-	-	-
FAP3	-	+	-	-	-	+	-	-	-	+	+	+
FAP4	-	+	-	-	-	+	-	+	-	-	-	+
FAP5	+	-	+	-	-	-	+	-	-	-	-	+
FAP6	+	-	+	-	-	-	+	-	-			

Sample Collection and Preparation:

Peripheral blood was collected from 6 Fanconi anemia patients from Andhra Mahila Sabha Hospital, Chennai, according to the Indian Council for Medical Research (ICMR) New Delhi, guidelines with the consent of the patients. All samples used for the study were characterized for the hypersensitivity to DNA damaging agent Mitomycin C by the clinician prior to sample collection. Peripheral blood was also collected from healthy individuals with no symptoms of FA who served as control for the current study. Samples were collected in sterile PAX gene Blood RNA tubes (PreAnalytix GmbH, Switzerland) and stored at -20˚C until further processing.

Total RNA was isolated from the samples using PAX gene blood RNA isolation kit according to the manufacturer's instructions (PreAnalytix GmbH, Switzerland). Total RNA was quantified by measuring the absorbance at 260 nm using a PerkinElmer Lambda 650 UV/VIS spectrophotometer (PerkinElmer, USA). RNA was amplified using Express Art mRNA amplification kit micro version (Artus GmbH, Germany). Amplified RNA was labeled with Cy3 Post-Labeling Reactive Dye Pack (GE Healthcare UK limited, UK). Labeled RNA was fragmented and purified using Express Art Amino allyl mRNA amplification kit and YM10 columns (Millipore, USA). 10.0 mg of the labeled amplified RNA was used for hybridization with the Human 40K (A+B) OciChip array. Hybridization was performed using automated hybstation HS 4800. Hybridized chips were scanned using Affymetrix 428TM array scanner at three different PMT gains. The interest was to determine probes (genes) that were differentially expressed (DE) between the normal and the FA samples. A threshold log fold change (LFC) of 3.0 was fixed to attain FDR of less than 0.05 and accordingly the DE genes were obtained.
